# Evaluation of compassionate and respectful care implementation status in model healthcare facilities: a cross-sectional study

**DOI:** 10.1186/s13690-022-00845-y

**Published:** 2022-03-16

**Authors:** Kemal Jemal, Assegid Samuel, Abiyu Geta, Fantanesh Desalegn, Lidia Gebru, Tezera Tadele, Ewnetu Genet, Mulugeta Abate, Nebiyou Tafesse

**Affiliations:** 1 Department of Nursing, College of medical and Health Sciences, Salale University, Fitche, Ethiopia; 2 Ministry of Health Ethiopia, Human Resource Development Directorate, Addis Ababa, Ethiopia; 3 Ethiopian Public Health Association, Addis Ababa, Ethiopia; 4 Department of Public Health, Menelik II Medical and Health Sciences College, Kotebe University of Education, Addis Ababa, Ethiopia

**Keywords:** Implementation, Compassionate, Respectful care, Health workforce, Ethiopia

## Abstract

**Background:**

Compassionate respectful, and caring (CRC) creates a pleasant environment for health workforce (HWF), customers, and families. For the past five years, the Ethiopian Ministry of Health (EMoH) has developed a CRC plan to improve person-centered care. Therefore, we aimed to assess the implementation status of CRC and associated factors in the 16 model health facilities (MHFs) in Ethiopia.

**Methods:**

A cross-sectional study was employed from February to April 2021. A structured and semi-structured questionnaire was used to assess the level of CRC implementation in model health care facilities. Epi-data version 4.3 and SPSS version 26 software were used for data entry and analysis, respectively. Binary logistic regressions analysis was used and significance was obtained at the odds ratio with a 95% confidence interval and *P*-value < 0.05.

**Results:**

A total of 429 HWF participated in a self-administered questionnaire. The prevalence of compassionate and respectful care among HWF were 60.4%, and 64% respectively. Nurse professionals, midwives, having training on CRC, leader promoting CRC, having a conducive working environment and burnout management for HWF were significantly associated with compassionate care practice. Leaders promoting CRC, having a conducive working environment, and burnout management for HWF were significantly associated with respectful care practice.

**Conclusion:**

The findings identified distinct issues related to CRC implementation in each 16 MHF. Addressing HWF skill gaps, a conducive working environment, and burnout management are encouraged CRC continuity. Incorporate CRC in pre-service education, health system strengthening, and motivating HWF are important for CRC strategic implementation.

## Background

Compassion is a feeling of deep sympathy and sorrow for the suffering of others accompanied by a strong desire to alleviate the suffering and/or being sensitive to the pain or suffering [1–3]. Compassionate care creates a healing link through understanding the patient's context and perspective, meeting the patient's intrinsic need, and guiding client decision-making [4, 5]. Compassion is associated with a number of values, including empathy, sympathy, kindness, and most importantly, the ability to care for others [6–8].

Respectful care is any type of care that supports and encourages a person's self-respect rather than undermining it, regardless of differences [9]. It has to do with paying attention, honoring, avoiding harm, not meddling or interrupting, treating others with respect, and accompanied by effective interventions to alleviate the suffering [10, 11].

The CRC is more important for person-centered care that HWF passionate about their profession and enjoys assisting others, being ethical, and being a model for young professionals and students [12, 13]. It serves as a foundation for medical ethics, a major source of flexibility, useful in forming connections, and critical in today's world for dealing with human rights [14]. Despite the fact that CRC is compulsory, HWF has been seen to skip it in favor of other areas of treatment. The CRC is a core stone for quality healthcare development [15], and plays a great role in health care facilities (HCFs) and community homeless services [16, 17].

According to research done in the Tigray region, Ethiopia, 44% of healthcare workers (HCWs) had a negative attitude about CRC [13]. In addition, a survey conducted in North Sowa Zone, Oromiya region found that 38.8% of HWF had provided good compassionate care and 46.2% had practiced respectful treatment [12]. Also in Ethiopia, an aggregated 3-year report (January 2011 to December 2013) by Health Professionals Ethics Federal Committee of Ethiopia indicated that 39 complaints were concerned with the patient's death, 15 complaints about impairment. One-fifth of the complaints regarding ethical breach and also transmitting harsh words, yelling at clients, maltreatment, insulting, and striking clients [18]. A similar seven-year (2011 to 2017) review analysis by Federal Ethics Committee For Health Professionals Ethics Review found that 57.6% of complaints were connected to mortality, and 21.6% were errors involving physical injury, ethical violation, and carelessness [19]. In addition, one-fourth of medical physicians were unaware of the code of ethics, and 39% of medical practitioners had negative opinions about the code of ethics in Ethiopia [20].

Compassion fatigue and burnout, mental and physical stress, decreasing attentiveness and empathy, workload and a demanding working environment, and a lack of enthusiasm for HCWs have all hampered the CRC [7, 21].

The CRC can help a person recover from a major illness, better manage chronic conditions, reduce client anxiety and stress, and is crucial for effective medical outcomes. Research evidence documented that Over 85% of clients and 76% of HCWs agreed on a positive medical outcome [22]. Psychological symbols and the type of HWF reaction may also have a role in CRC advance, offering fresh insights into the treatment interaction and boosting attitudes toward behavioral changes [1, 23].

Many professionals in Ethiopia are sympathetic and aware of the abilities that are required for CRC. Conversely, many healthcare practitioners do not provide CRC to clients or their families [24]. Ethiopian government has been implementing the CRC program and attempts to improve person-centered care during the last five years to close these disparities. Respect for clients' fundamental human rights, autonomy, dignity, sentiments, desires, and choice of friendship must all be respected wherever possible. Therefore, this study aimed to evaluate the CRC implementation in 16 MHFs in Ethiopia. The findings of this study may be useful to Sub-Saharan African countries and literature evidence for scholars by demonstrating the actual figure and factors affecting CRC, as well as the future outlook for CRC implementation scalability.

## Methods

### Study design, area, and period

This nationwide cross-sectional study was conducted in 16 MHFs from February to April 2021. The 16 MHFs were previously selected as CRC incubation centers starting from 2015. In June 2020, Ethiopia has more than 273,601 HWF employed in public health facilities. Around 181,872 (66.5%) are health professionals and the remaining 91,723 (33.5%) are administrative staff [25].

Among health professionals, the highest three professional categories are Nurses (59,063 (32.5%)), Health Extension Workers (41,826 (23%)), and Midwifery (18,350 (10.08%). The private health sectors provide work opportunities for about 60,000 human resources for health in Ethiopia. There are 17,162 functional health posts 3,678 health centers and 340 all types of hospitals across all regions of the country that provide health services to the community. There are 22 tertiary hospitals, 73 general hospitals, and 245 primary hospitals in the year 2020 [25].

### Participant institutes

From the nine regions and two city administrations, all 16 MHFs that had already been recruited for CRC implementation were selected. Due to the bigger population and numerous MHFs located in Addis Ababa, five MHFs from the city administration of Addis Ababa and one from the city administration of Dire Dewa were involved. From each of the eight regions, one MHF was chosen (Afar, Amhara, Oromia, Gambela, Sidama, Harari, Benshangul-Gumuzi, and Somali). Two MHFs were selected from South Nation Nationality People (SNNP) (Arbaminch General Hospital and G/tsadik Shawa). The HWF who have been working for more than six months in 16 MHFs in Ethiopia were included in the study. The HWF who have been on annual leave and transferred from other health care facilities that served less than six months were excluded from the study. According to WHO definition HWF can be defined "all people (clinical staff, management and support staff, managers, ambulance drivers and accountants) engaged in actions whose primary intent is to enhance health" [26].

### Sample size determination and sampling procedure

The sample size was determined by using Epi Info version 7 software [27] by single population proportion formula with the assumptions of a 95% confidence level, and a 5% precision, taking 50% proportion due to the lack of the previous study. The sample size of 435 was obtained after adding a 13% non-response rate. The HWF from the 16 MHFs were selected using a simple random sampling technique from the list of each healthcare department. Proportional allocation was used based on the number of the HWF per each 16 MHF.

### Data collection tools and procedure

Data were collected using a standardized and pre-tested questionnaire. The questionnaire was adapted from different works of literature and the Ethiopian HWF training participant manual for CRC [28, 29]. The questionnaire contains socio-demographic characteristics, previous training on CRC, types of health facilities, and facilities auditing (an observational checklist). Observational checklist were administered to Chief Executive Director, Quality Director and Medical Director, and observed the CRC implementation guideline and manual, independent CRC plan, office and focal person, CRC discussed minutes, list of CRC committee and documentation of best practices finding from the 16 MHFs (Table 4).

The questionnaire was prepared in the English language, translated into Amharic, Afan Oromo, Somali language, and back-translated to English language-by-language experts. Data were collected using a self-administration questionnaire for the health workforce. Data were collected by 34 data collectors and supervised by 17 supervisors. Data collectors and supervisors were recruited based on their ability to speak Amharic language and fluent in each specific region language for he/she recruited for data collection and supervision in the regions with having previous experience of data collection.

Data collectors and supervisors were trained for five days on the study's purpose, details of the questionnaire, interviewing techniques, the importance of privacy, and ensuring the respondents' confidentiality. In addition to data collection training, COVID-19 prevention protocol training was provided for data collectors and supervisors. The pre-test was done in non-MHFs other than the study area having the same socio-demographic characteristics. The questionnaire was reviewed and checked for completeness, accuracy, and consistency by the supervisor and principal investigator to take timely corrective measures.

The implementations of compassionate and respectful care were measured using a five-point Likert scale from 1 to 5 (1 = almost never, 2 = seldom, 3 = sometimes, 4 = often, 5 = almost always). The tools contain 24 items for compassionate care and 21 items for respectful care. Taking the mean scores as a cut-off point, the outcomes of compassionate and respectful care were calculated after testing each outcome’s results’ normality distribution. The scores greater than the mean score were considered compassionate care and respectful care. Below the mean scores were considered poor compassionate and disrespectful care. The CRC instruments have been previously demonstrated and verified in the Ethiopian language context [12, 30]. In addition, the questionnaires’ reliability was checked in a pre-tested questionnaire with Cronbach’s alpha 0.66 for compassionate care and 0.72 for respectful care.

### Data processing and analysis

Data were coded, edited, cleaned, and entered into Epi-data version 4.3 and transported to SPSS version 26. The descriptive data analysis was done and presented in frequency, summary statistics, tables, and graphs. The outcome variables were dichotomized based on the cut-off point of the mean for binary logistic regression. Variables with *P*-value ≤ 0.2 in the binary analysis were included in a multivariable logistic regression analysis to control the confounding effect among the variables. Statistical significance was declared if *P*-value < 0.05.

### Ethical consideration

Ethical clearance was obtained from the Ethiopian Public Health Association on February 04/2021 Ref.No. EPA04/048/21. Written permission was obtained from the 16 MHFs. Informed written consent was obtained from each respondent. The confidentiality of respondents was maintained by excluding their names from the questionnaire. The respondents were informed that their inclusion in the study is voluntary, and are free to withdraw from the study if not willing to participate.

## Results

In the study, 429 HWF participated with a response rate of 98.6%. The majority of the study participants were women with 51.7%, and 39.9% of the study participants were aged between 25 to 29 years old. In relation to the educational status, 61.1% were bachelor holders, and nurses account for 37.5%. Regarding the work experience, 40.1% of them have 5 to 10 years of experience (Table 1).

**Table 1:** socio-demographic of HWF at 16 MHFs in Ethiopia 2021(*n* = 429)

Variables	number of study participants	Percentage
**Age**		
20 -24	29	6.8
25–29	171	39.9
30–34	123	28.7
35–39	52	12.1
≥ 40	54	12.6
**Sex**		
Male	207	48.3
Female	222	51.7
**Educational status**		
Diploma	63	14.7
BSC/ MD	262	61.1
MSC	78	18.2
Specialty/PhD	26	6.1
**Current profession**		
Medical doctor	68	15.9
Public health	26	6.1
Nurse	161	37.5
Midwife	43	10
Anesthesia	36	8.4
Laboratory	28	6.5
Pharmacy	52	12.1
Others	15	3.5
**Working department**		
OPD	83	19.3
Surgery	67	15.6
GYN	62	14.5
Radiology	107	24.9
Laboratory	27	6.3
Pharmacy	22	5.1
Emergency	50	11.7
Others	11	2.6
**Working experience**		
< 5 years	164	38.2
5 -10 years	172	40.1
> 10 years	93	21.7
**Have trained on CRC**		
Yes	234	54.5
No	195	45.5
**The leader promoting CRC implementation**		
Yes	258	60.1
No	171	39.9
**Have a conducive working environment**		
Yes	240	55.9
No	189	44.1
**Burnout management for HWFs is important**		
Very important	361	84.1
Less important	68	15.9

### Compassionate care

**Fig. 1:**
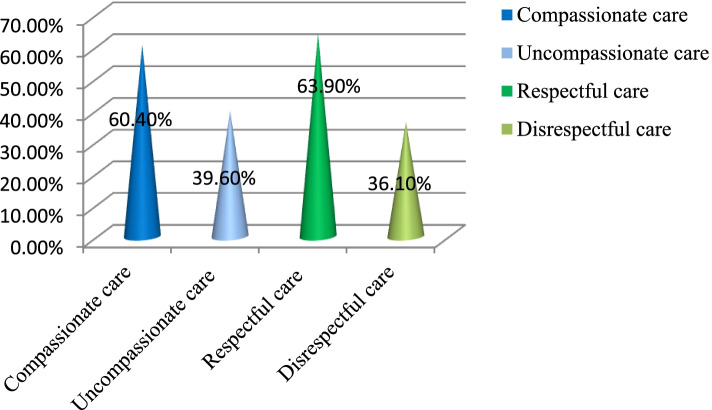
The prevalence of compassionate and respectful care among HWF at 16 MHFs in Ethiopia, 2021 (*n* = 429)

The prevalence of compassionate care among HWF in 16 MHFs was 60.4% (Fig. 1). During health care service delivery, 51.5% of health professionals introduce themselves to their clients, and 66% of HWF called their clients by their names. Besides, 73.9% of health professionals engaged themselves in conversation with clients. In line with that, 82.5% were actively listening, 80% were shown love and tolerance, 83.9% understood client needs, and 76.5% understood their clients' emotions (Table 2).

**Table 2:** Compassionate care practice among HWF at 16 MHFs in Ethiopia, 2021 (*n* = 429)

Variables	Number of study participants	Percentage
Healthcare providers introduce themselves properly		
Yes	221	51.5
No	208	48.5
Do you call the client by their names		
Yes	283	66.0
No	146	34.0
Engage themselves with the client conversation		
Yes	317	73.9
No	112	26.1
Actively listed what the client is said		
Yes	354	82.5
No	75	17.5
Show love and tolerance to the clients		
Yes	343	80.0
No	86	20.0
Try to understand the clients' need		
Yes	360	83.9
No	69	16.1
Actively understand the clients’ emotions		
Yes	328	76.5
No	101	23.5
Use probing and supportive words		
Yes	289	67.4
No	140	32.6
Respond promptly and professionally when the client needs		
Yes	346	80.7
No	83	19.3
Involved in client treatment options and decision-making process		
Yes	312	72.7
No	117	27.3
Frequently communicate and collaborate with the healthcare team		
Yes	361	84.1
No	68	15.9
Have trained on CRC		
Yes	234	54.5
No	195	45.5
Did you practice CRC principles in your facility		
Yes	223	52.0
No	206	48.0
The leader promoting CRC implementation		
Yes	258	60.1
No	171	39.9

To understand the clients well, 67.4% of health professionals use probing and supportive words and 80.7% of the professionals respond promptly and professionally to clients' concerns and questions. As part of client empowerment and informed decision-making, 72.7% of health professionals involve clients in decision-making and treatment options. Along with that, for the better outcome of the health care service, 84.1% of practitioners frequently collaborate with the health care team. In these study settings, 54.5% of the respondent has taken training on CRC. The study participants also believed that leaders of the facilities promote and influence the implementation of CRC (60.1%) (Table 2).

### Respectful care

The prevalence of respectful care among the HWF was 64% in 16 MHFs (Fig. 1). In day-to-day health care service delivery, 77.6% of health professionals greet their clients with respect, and 52% introduce themselves. Around 72% of health professionals effectively address clients' issues by considering their age and social status (Table 3).

**Table 3:** Respectful care practice among HWF at 16 MHFs in Ethiopia, 2021(*N* = 429)

Variables	Participants	Percentage
Do you greet the client respectfully		
Yes	333	77.6
No	96	22.4
Do HWF introduce themselves to the clients		
Yes	223	52.0
No	206	48.0
Properly address clients considering their social status and age		
Yes	308	71.8
No	121	28.2
Actively listen to clients		
Yes	337	78.6
No	92	21.4
Allocate adequate time for the client to talk		
Yes	324	75.5
No	105	24.5
Respect patient’s view on treatment and care		
Yes	344	80.2
No	85	19.8
Obtain consent before examination and procedures		
Yes	329	76.7
No	100	23.3
Ensure confidentiality of patient information		
Yes	362	84.4
No	67	15.6
Maintain privacy in providing clinical care		
Yes	351	81.8
No	78	18.2
Do HWF in your health facility verbally abuse clients		
Yes	115	26.8
No	314	73.2
Treat clients equally without discrimination		
Yes	322	75.1
No	107	24.9
Responds promptly and professionally when clients ask for help		
Yes	357	83.2
No	72	16.8
Give adequate information regarding patient treatment and care		
Yes	325	75.8
No	104	24.2
In your health facility, clients physically abused (slapping, pinching, restraint…)		
Yes	128	29.8
No	301	70.2
In your health facility abandon patients without care for a long time		
Yes	180	42.0
No	249	58.0
Have good communication and collaboration within the team		
Yes	333	77.6
No	96	22.4
Do the guards in your health facility receive patients and families with respect		
Yes	295	68.8
No	134	31.2
Do the record officers treat patients and families with respect		
Yes	287	66.9
No	142	33.1
Does your facility detain clients without their will		
Yes	167	38.9
No	262	61.1
Does your facility ensure a safe and clean care environment for clients?		
Yes	240	55.9
No	189	44.1

About 78.6% of the respondents actively listen, and 75.5% believed to provide adequate time for clients to talk and raise their concerns. Along with attentive and considerate care, 80.2% of the respondent’s respect patient views on treatment and care. More than three fourth (76.7%) of the respondents ensure obtaining consent before any examination and procedures, 84.4% of the respondents ensure confidentiality of patient information, and 81.8% maintain privacy during clinical care. Given the fact that health professionals respect their clients in many aspects, however, 26.8% of the respondents abuse clients verbally (Table 3).

In relation to justice, 75.1% of the respondents provide service without discrimination, and 83.2% were highly responsive when clients seek help, likewise, 75.8% provide ample information concerning patient treatment and care. Contrary to the provision of fair and respectful care, 29.8% of health facilities breached justice (clients abused physically), 42% rate abandonment of care for a long time and 38.9% detained clients irrespective of their will (Table 3).

Within the health care team, 77.6% of the respondents have good communication with each other. In addition, 68.8% of the guards, and 66.9% of the record officers of the facilities welcomed and take care of clients and their families with respect. Furthermore, 55.9% of the health care facilities ensured a safe and clean environment for clients (Table 3).

### Observational checklist

The implementation of CRC was found to be different from facility to facility. About 87.5% has an independent plan on CRC, however, only 18.8% of the facilities allocated finance for CRC implementation. In relation to facility leadership, 87.5% of the facilities have board members to observe the facility, and 56.3% of board meetings discussed CRC as an agenda. Three-fourth of the facilities management members took the agenda of CRC as a priority topic of discussion, and 93.8% assigned CRC focal persons (Table 4).

**Table 4:** Observational checklist for CRC at 16 MHFs in Ethiopia, 2021 (*n* = 16)

Variable	N(%)	Types of facilities		
		General Hospital	Specialized	Tertiary
have an independent plan on CRC				
Yes	14(87.5)	9(100)	3(75)	2(66.7)
No	2(12.5)	0(0)	1(25)	1(33.3)
have an annual allocated financial plan for CRC				
Yes	3(18.8)	2(22.2)	1(25)	0(0)
No	13 (81.3)	7(77.8)	3 (75)	3(100)
have a board for the leadership of the facility				
Yes	14(87.5)	8(88.9)	4(100)	2(66.7)
No	2(12.5)	1(11.1)	0(0)	1(33.3)
Raised agenda of CRC on board meeting				
Yes	9(56.3)	5(55.6)	3(75)	1(33.3)
No	7(43.8)	4(44.4)	1(25)	2(66.7)
Management discussed CRC				
Yes	12(75)	7(77.8)	3(75)	2(66.7)
No	4(25)	2(22.2)	1(25)	1(33.3)
Have currently CRC focal persons				
Yes	15(93.8)	9(100)	3(75)	3(100)
No	1(6.3)	0(0)	1(25)	0(0)
CRC focal person has an independent office				
Yes	5(31.3)	2(22.2)	2(50)	1(33.3)
No	11(68.8)	7(77.8)	2(50)	2(66.7)
Availability of the CRC manual per each department				
Yes	5(31.3)	2(22.2)	1(25)	2(66.7)
No	11(68.8)	7(77.8)	3(75)	1(33.3)
Does the facility have a CRC committee				
Yes	13(81.3)	6(66.7)	4(100)	3(100)
No	3(18.8)	3(33.3)	0(0)	0(0)
Facility select the CRC ambassador				
Yes	14(87.5)	7(77.8)	4(100)	3(100)
No	2(12.5)	2(22.2)	0(0)	0(0)
Have regular selection and announcement of model CRC performer				
Yes	13(81.3)	7(77.8)	4(100)	2(66.7)
No	3(18.8)	2(22.2)	0(0)	1(33.3)
Have community forums and discussions on CRC				
Yes	10(62.5)	5(55.6)	3(75)	2(66.7)
No	6(37.5)	4(44.4)	1(25)	1(33.3)
Have a recent meeting with community representatives on CRC				
Yes	14(87.5)	8(88.9)	4(100)	2(66.7)
No	2(12.5)	1(11.1)	0(0)	1(33.3)
Have a voluntary service on CRC				
Yes	10(62.5)	5(55.6)	3(75)	2(66.7)
No	6(37.5)	4(44.4)	1(25)	1(33.3)
Have an MOU plan in CRC with related stakeholders				
Yes	10(62.5)	4(44.4)	4(100)	2(66.7)
No	6(37.5)	5(55.6)	0(0)	1(33.3)
Table continued				
Have a conducive working environment				
Yes	8(50)	4(44.4)	2(50)	2(66.7)
No	8(50)	5(55.6)	2(50)	1(33.3)
Implement CRC with a quality strategy				
Yes	11(68.8)	6(66.7)	2(50)	3(100)
No	5(31.3)	3(33.3)	2(50)	0(0)
Have a skill lab or CRC simulation center				
Yes	12(75)	7(77.8)	4(100)	1(33.3)
No	4(25)	2(22.2)	0(0)	2(66.7)
CRC include in health education provision				
Yes	7(43.8)	5(55.6)	2(50)	0(0)
No	9(56.3)	4(44.4)	2(50)	3(100)
Include CRC in the morning session discussion				
Yes	7(43.8	2(22.2)	2(50)	3(100)
No	9(56.3)	7(77.8)	2(50)	0(0)
Does the facility select model units in CRC per department				
Yes	7(43.8	2(22.2)	3(75)	2(66.7)
No	9(56.3)	7(77.8)	1(25)	1(33.3)
Have a system to treat the HWFs with compassionate care				
Yes	10(62.5)	5(55.6)	2(50)	3(100)
No	6(37.5)	4(44.4)	2(50)	0(0)
Have ways to manage burnout and motivation mechanisms for HWFs				
Yes	7(43.8)	4(44.4)	2(50)	1(33.3)
No	9(56.3)	5(55.6)	2(50)	2(66.7)
Provide induction orientation on CRC for newly recruited staff				
Yes	12(75)	6(66.7)	3(75)	3(100)
No	4(25)	3(33.3)	1(25)	0(0)
Assess the performance or implementation of CRC in the facility				
Yes	11(68.8)	6(66.7)	3(75)	2(66.7)
No	5(31.3)	3(33.3)	1(25)	1(33.3)
IST center integrate CRC packages in the continuous professional development				
Yes	10(62.5)	4(44.4)	4(100)	2(66.7)
No	6(37.5)	5(55.6)	0(0)	1(33.3)
Does the facility recently introduce the best practice on CRC?				
Yes	9(56.3)	4(44.4)	3(75)	2(66.7)
No	7(43.8)	5(55.6)	1(25)	1(33.3)
Is /are there challenges and opportunities during the implementation of CRC?				
Yes	10(62.5)	6(66.7)	3(75)	1(33.3)
No	6(37.5)	3(33.3)	1(25)	2(66.7)
Work with the nearby pre-service education, elementary and high school				
Yes	5(31.3)	3(33.3)	2(50)	0(0)
No	11(68.8)	6(66.7)	2(50)	3(100)

Despite the fact that many facilities dedicated, only 31.3% of them have an independent office for CRC focal person. The CRC materials are also not widely accessible, only 31.3% of the facilities have CRC implementation guidelines and training manuals. About 81.3% of the facilities had established CRC committees, and 87.5% of the facilities were selected CRC ambassadors that selected from role model HWF, and has made advocacy and mentor others to implement CRC. To ensure the sustainability of CRC implementation, 81.3% of the facilities were selected and introduced best CRC performers (Table 4).

As part of community engagement, 62.5% of the facilities have organized community forums on CRC, and 87.5% had a recent meeting with the community representatives. Additionally, 62.5% of facilities have organized volunteerism and signed a memorandum of understanding with stakeholders. In relation to the working environment, only 50% of the facilities have a conducive working environment (that promotes employee and client safety, comfortable and clean physical space, availability of adequate resources to provide quality health services and better feedback atmosphere both for clients and health workforce). More than two-third (68.8%) of the facilities were integrated CRC implementation with quality strategy (Table 4).

### Factors associated with compassionate and respectful care practice

In bivariate logistic regression analysis, female participants, nurse and midwife professionals, having training on CRC, leaders promote the CRC in their institution, having conducive working environment and burnout management for HWFs were significantly associated while female participants were adjusted for compassionate care practice in multivariable logistic regression (Table 5). Likewise, female participants, having BSC educational level, nurse professionals, leaders promote the CRC in their institution, having conducive working environment and burnout management for HWF were significantly associated with respectful care whereas female participants, having BSC educational level, and nurse professionals were adjusted in multivariable logistic regression analysis for respectful care practice (Table 6).

**Table 5:** Factors (crude and adjusted odds ratios, confidence intervals, and p-value) associated with compassionate care among HWF at 16 MHFs in Ethiopia (*n* = 429)

Variables	Compassion		Crude OR (95% CI)	*p*-value	Adjusted OR (95% CI)	*p*-value
	Yes	No				
Age						
20–29	120	80	1		1	
30–39	105	70	1.01(0.66,1.51)	0.693	0.85(0.61,1.99)	0.757
≥ 40	34	20	1.13(0.61,2.11)	0.207	0.99(0.42,2.30)	0.977
Sex						
Male	109	98	1		1	
Female	150	72	1.87(1.27,2.77)	**0.002**	1.49(0.91,2.43)	0.110
Educational status						
Diploma	40	23	1.43(0.76,2.73)	0.271	1.21(0.46,3.18)	0.693
BSC	162	100	1.34(0.84,2.12)	0.217	0.66(0.31,1.39)	0.277
MSC and above	57	47	1		1	
Profession						
Nurse	119	42	5.98(2.51,14.24)	**0.001**	4.16(2.21.9.38)	**0.001**
Medical doctor	36	32	2.38(0.94,5.99)	0.067	2.21(0.68,4.22)	0.190
Public health officer	20	21	2.01(0.74,5.48)	0.172	1.98(0.80,4.93)	0.152
Midwifes	30	13	4.87(1.75,13.59)	**0.002**	3.31(1.60,8.62)	**0.006**
Anesthesia	19	17	2.36(0.84,6.60)	0.102	2.07(0.80,4.93)	0.109
Pharmacy	26	26	2.11(0.81,5.52)	0.128	1.76(0.98,3.38)	0.052
Others	9	19	1		1	
Working experience						
< 5	97	67	1		1	
≥ 5	162	103	1.09(0.73,1.62)	0.683	0.73(0.39,1.36)	0.320
Trained on CRC						
Yes	173	61	3.60(2.39,5.40)	**0.001**	2.75(1.67,4.53)	**0.001**
No	86	109	1		1	
Leader promoting CRC						
Yes	186	72	3.47(2.31,5.21)	**0.001**	2.34(1.42,3.87)	**0.001**
No	73	98	1	1	1	
Conducive working environment						
Yes	165	75	2.22(1.50,3.30)	**0.001**	1.70(1.05,2.74)	**0.031**
No	94	95	1		1	
Burnout management for HWF is important						
Very important	247	114	10.11(5.22,19.60)	**0.001**	6.92(3.31,14.44)	**0.001**
Less important	12	56	1		1

**Table 6:** Factors (crude and adjusted odds ratios, confidence intervals, and p-value) associated with respectful care among HWF at 16 MHFs in Ethiopia (*n* = 429)

Variables	Respectful care		Crude OR (95% CI)	*p*-value	Adjusted OR (95% CI)	*p*-value
	Yes	No				
Age						
20–29	126	74	1		1	
30–39	109	66	0.97(0.64,1.48)	0.887	0.85(0.47,1.56)	0.598
≥ 40	39	15	1.53(0.79,2.96)	0.209	1.25(0.50,3.10)	0.637
**Sex**						
Male	115	92	1		1	
Female	159	63	2.02(1.35,3.01)	**0.001**	1.33(0.81,2.21)	0.261
Educational status						
Diploma	40	23	1.49(0.79,2.83)	0.223	0.84(0.32,2.19)	0.716
BSC	178	84	1.82(1.14,2.89)	**0.012**	0.79(0.38,1.67)	0.544
MSC and above	56	48	1		1	
Profession						
Nurse	119	42	2.46(1.08,5.59)	**0.032**	2.19(0.75,6.35)	0.151
Medical doctor	36	32	0.98(0.40,2.36)	0.955	0.75(0.23,2.51)	0.644
Public health officer	26	15	1.50(0.57,3.99)	0.415	1.41(0.44,3.19)	0.510
Midwifes	31	12	2.24(0.83,6.07)	0.113	1.96(0.56,5.82)	0.291
Anesthesia	21	15	1.21(0.45,3.28)	0.761	1.19(0.37,4.50)	0.687
Pharmacy	26	26	0.87(0.35,2.18)	0.761	0.84(0.29,2.03)	0.921
Others	15	13	1		1	
Working experience						
< 5	99	65	1		1	
≥ 5	175	90	1.28(0.85,1.91)	0.235	0.99(0.53,1.83)	0.967
Trained on CRC						
Yes	159	75	1.48(0.99,2.19)	0.054	0.77(0.45,1.31)	0.332
No	115	80	1		1	
Leader promoting CRC						
Yes	193	65	3.30(2.19,4.98)	**0.001**	2.55(1.52,4.29)	**0.001**
No	81	90	1	1	1	
Conducive working environment						
Yes	199	41	7.38(4.73,11.51)	**0.001**	6.94(2.24,9.38)	**0.001**
No	75	114	1		1	
Burnout management for HWF is important						
Very important	255	106	6.20(3.49,11.04)	**0.001**	4.29(2.18,8.44)	**0.001**
Less important	19	49	1		1	

In multivariable logistic regression analysis, nurse professionals [AOR = 4.16; 95% CI = (2.21,9.38)], midwifes [AOR = 3.31; 85% CI = (1.60,8.62)], having training on CRC [AOR = 2.75; 95% CI = (1.67,4.53)], leader promoting CRC in the health facilities [AOR = 2.34; 95% CI = (1.42,3.87)], having conducive working environment in the health care facilities [AOR = 1.70; 95% CI = (1.05,2.74)], and burnout management for HWF [AOR = 6.92; 95% CI = (3.31,14.44)] were significantly associated with compassionate care among HWF at 16 MHFs in Ethiopia (Table 5).

Regarding respectful care, leaders who promote CRC in the health care facilities were 2.55 times more likely to practice respectful care than the leaders who did not promote CRC in the health facilities [AOR = 2.55; 95% CI = (1.52,4.29)]. Having a conducive working environment in health care facilities were seven times more likely to practice respectful care when comparing with those who have no conducive working environment in health care facilities [AOR = 6.94; 95% CI = (2.24,9.38)]. Burnout management for HWF was four times more likely to practice respectful care when compared with health care facilities that had not burnout management for their HWF [AOR = 4.29; 95% CI = (2.18,8.44)] (Table 6).

## Discussion

The current result revealed that the CRC practice have been showing progress in 16 MHFs. Furthermore, we identified that have training on CRC, leaders who have promoted CRC implementation, having a conducive working environment, and burnout management are positive indicator for CRC progress.

We found that the prevalence of compassionate and respectful care was 60.4% with a 95% confidence interval of (55.9% to 64.6%) and 63.9% with a 95% confidence interval of (59.2% to 68.1%), respectively. This result was higher than the previous study finding in non-MHFs in the North Showa Zone, which found 38.8% for compassionate care and 46.2% for respectful care practice [12]. This discrepancy is possible because in this study all MHF were trained by the Ethiopian Ministry of Health and each Regional Health Bureau. On the other hand, our result was lower than the study reported in Northwest Ethiopia that found 71.8% of compassionate care and 82.6% respectful care among outpatient clients [30]. Several studies have found that the CRC is vital for better adherence to medical advice and treatment plans, faster healing processes, better clinical outcome, improve health care system and reduce malpractice [16, 31, 32].

In the current study, we have found that nurses and midwives were significantly associated with better provision of compassionate care. A meta-analysis study documented that self-compassion interventions have an impact on improving compassion, respectful care, self-compassion, and mindfulness for health care professionals [33].

We found that the study participants who have training on CRC have significantly associated with compassionate care. Various studies confirmed that having training can assist healthcare professionals to increase mental health resilience, improve patient care, and minimize burnout [34, 35]. The CRC training is an important first step toward further updating the caring, patient right, and responsibility of HWF competency. The HWF employed in MHFs had obtained CRC training, however, HWF may turnover time to time. The new employed HWF may need orientations to enhance their knowledge, perception, and practice of CRC in the MHFs.

In this study, 60.1% of the leaders have promoted CRC implementation in the 16 MHFs. This finding is similar to a previous study report in non-MHFs [12]. This indicated that the implementation of CRC in 16 MHFs has no progress on leadership and managers. The components of good care including, improved quality, increased productivity, nurtured compassion, ensured effectiveness, stimulated innovation, and maintained patient satisfaction can only be achieved when leaders are compassionated [36]. As strategies to sustainable CRC in the health care facilities, improving health care system strengthening and developing compassionate and innovative leader are important to inspire with genuine team collaboration across professional boundaries.

Having a conducive working environment was associated with compassionate and respectful care practice. Employees expect health care facilities to provide a model-working environment that promotes efficiency, effectiveness, production, luxury, and job dedication [37]. Conversely, an inadequate working environment has an impact on health care professionals' performance, the quality of health care delivery, and the practice of client-centered and compassionate care [38].

To sustain an appropriate HWF, it is critical to creating a healthy working environment for clinical care practice. The demanding nature of the occupation frequently leads to burnout, disability, and high absenteeism, all which contribute to fatigue for compassionate care [39]. The Ministry of Health plays a critical role in HWF retention by establishing a conducive healthcare environment to create high-quality results for both staff and clients.

Burnout management for HWF was significantly associated with compassionate and respectful care practice. A study done in Kenya reported similar findings that sufficient career training, job security, salary, supervisor support, and manageable workload, family health care insurance, and terminal benefits were identified as motivation and reward of HWF retention mechanisms [40]. Due to a lack of trained human resource, HWF in Ethiopia often reported on taking additional responsibility that adds to duties for which they lack the necessary skills and training.

The results of this study show the implementation of CRC in MHFs are important implication for enhancing CRC program in low resource countries, which was helpful for understanding of the CRC implementation and promote scalability of the CRC program into non-MHFs. Moreover, the results are important inputs to researchers, policymakers, social workers, clinicians, public health promoters, and community leaders to prioritize CRC implementation in their planned activities.

### Strength and limitation of the study

This study used both an observational checklist and self-administered questions to strengthening the finding more for a better outcome. In addition, the study was conducted at national level and is thus representative of all regions of Ethiopia that selected as CRC model health care facilities.

As a limitation, the study utilizes the cross-sectional study design that not determines the causality. The study participants are not homogeneous; they are all members of the health workforce, which included in the study who work in healthcare facilities and have varying levels of knowledge and skill. Finally, we had carefully outlined clear and short self-administered questionnaire, even still might be a response bias.

## Conclusion

The findings revealed a variety of challenges with CRC implementation in each of the 16 MHFs. Have training on CRC, leaders who have promoted CRC implementation, having a conducive working environment, and burnout management for HWF are all strong predictors of CRC. Therefore, incorporating in pre-service education, advocacy and system strengthening, and motivating HWF are the most sustainable measures. In addition, the EMoH should invest in the expended implementation of MHFs for all health-care facilities in order to strengthen Ethiopia's HWF and health-care systems.

## Data Availability

The datasets generated and/or analyzed during the current study are not publicly available. Sharing of data was not included in the approval from the ethics committee but is available from the corresponding author on a reasonable request.

## Abbreviations

CRC: Compassionate and respectful care
EMoH: Ethiopian Ministry of Health
HCFs: Health care facilities
HCWs: Health care workers
HWF: health workforce
MHFs: Model health facilities
